# Community-based conservation reduces sexual risk factors for HIV among men

**DOI:** 10.1186/1744-8603-9-27

**Published:** 2013-07-09

**Authors:** Robin Naidoo, Kiersten Johnson

**Affiliations:** 1 Conservation Science Program, WWF-US, 1250 24th Street, Washington, DC 20037, USA; 2 Institute for Resources, Environment, and Sustainability, University of British Columbia, 2202 Main Mall, Vancouver, BC V6T 1Z4, Canada; 3 Demographic and Health Surveys, ICF International, 11785 Beltsville Dr Suite 300, Calverton, MD 20705, USA

**Keywords:** HIV, Primary prevention, Environment and public health, Community-based natural resource management, Biodiversity conservation

## Abstract

**Background:**

Despite numerous programs to combat the global HIV and AIDS pandemic, infection rate*s* remain high, especially in sub-Saharan Africa, where two-thirds of all people living with HIV reside. Here, we describe how we used rigorous program evaluation methods to assess the effectiveness of a community-based natural resource management program that “mainstreamed” HIV awareness and prevention activities within rural communities in Namibia.

**Findings:**

We used data from two rounds of the Namibia Demographic and Health Surveys (2000 and 2006/2007) and quasi-experimental statistical methods to evaluate changes in critical health-related outcomes in men and women living in communal conservancies, relative to several non-conservancy comparison groups. Our final dataset included 117 men and 318 women in 2000, and 170 men and 357 women in 2006/2007. We evaluated the statistical significance of the main effects of survey year and conservancy residence, and a conservancy-year interaction term, using generalized linear models. Our analyses show that community-based conservation in Namibia has significantly reduced multiple sexual partnerships, the main behavioural determinant of HIV/AIDS infection in Africa.

**Conclusions:**

Our results demonstrate the effectiveness of holistic community-based approaches centered on the preservation of lives and livelihoods, and highlight the potential benefits of integrating conservation and HIV prevention programming in other areas of communal land tenure in Africa.

## Findings

Although HIV and AIDS is a global pandemic, two-thirds of all people living with HIV (22.5 million) reside in sub-Saharan Africa. The epidemic appears to have stabilized, but the rate of new infections remains high and HIV continues to devastate families and communities, despite numerous programmatic approaches across the globe to combat the disease [1]. Here, we use rigorous program evaluation methods [2] to show that a national community-based conservation program in Namibia has significantly reduced multiple sexual partnerships, the main behavioural determinant of HIV infection in Africa.

Namibia’s Community-Based Natural Resources Management (CBNRM) program began in the 1990s and has coincided with increases in wildlife numbers and incomes in communal ‘conservancies’, i.e., customary landholdings having plans for zoning and sustainable use of natural resources [3,4]. From 2003–2007, PEPFAR (The United States President’s Emergency Plan for AIDS Relief), part of the United States’ Global Health Initiative, funded a community-based HIV/AIDS outreach and education program that was “mainstreamed” in 31 conservancies. Mainstreaming is a process allowing development actors to effectively and sustainably address the causes and effects of HIV and AIDS within the contexts of the normal functioning of an organization or a community [5]. Indeed, earlier Global Health Initiatives had already recognized the importance of engaging community-based organizations to combat HIV and AIDS in Africa [6]. In Namibia, the mainstreamed CBNRM HIV/AIDS program made explicit the links between HIV prevention and maintenance of conservancy-based livelihoods, and leveraged existing governance and management structures in conservancies to engage in culturally-appropriate prevention activities and behavior-change communication. Specifically, this holistic program raised awareness of the disease through radio broadcasts, written material, and traditional song and dance; trained peer educators; drafted HIV policies and plans; and disseminated condoms [7]. The focus in the 2003–2007 phase of the program that we evaluated was on “ABC” (Abstain, Be faithful, and use Condoms), as well as increasing access to treatment, support, and health care. In addition to community-level work, capacity, policies, and support activities were enhanced within national-level CBNRM support organizations.

## Methods

We used Demographic and Health Surveys (DHS) data from 2000 and 2006/2007 to evaluate whether changes in numbers of sexual partners were related to exposure of rural Namibians to the community-based HIV/AIDS program [8]. As part of a nationally-representative sampling scheme for women and men aged 15–49, DHS surveys included 204 households in 8 conservancies in 2000, and 259 households in 10 conservancies in 2006/07. While DHS data are globally recognized and utilized in the development of public health policy [9], we acknowledge the limitations of using self-reported data on the number of sexual partnerships, especially where interventions of the type we consider may lead to underreporting. Any such’social desirability bias’ is expected to be greater among women that among men [10]. DHS surveys in Namibia were a collaborative effort of the Namibia Ministry of Health and Social Services, the Central Bureau of Statistics, and the MEASURE DHS project of ICF Macro. Survey design and implementation passed review from a national Steering Committee, a national ethics review panel, and the ICF Macro Institutional Review Board. Informed consent was obtained from all survey respondents, participation was voluntary, and no compensation was provided.

To evaluate program impact over this time period, we compared trends in conservancies with three non-conservancy comparison groups: (1) all men/women outside of conservancies; (2) all men/women in the nearest DHS sampling cluster outside of each surveyed conservancy; and (3) a matched comparison group from quasi-experimental statistical matching [11] that was similar to conservancy residents in terms of characteristics that might confound program impact.

For the quasi-experimental comparison group (3), conservancy men and women were (separately) matched with men and women outside of conservancies using the following variables from 2000:

(1) Marital status

(2) Age

(3) Wealth quintile

(4) Education level

(5) Urban/rural residence

(6) Religious affiliation

(7) Distance to the nearest health clinic

(8) Geographical (administrative) region

(9) Precipitation

(10) Altitude

Variables (1) and (2) are standard indicators of exposure to sexual intercourse; variables (3) - (5) are correlates of higher-risk sexual behaviour in adults (e.g. [12,13]), and we further controlled for other potential social differences (6), access to health care (7), and broader structural and environmental differences that may affect disease transmission and proxy for unobserved effects on health and sexual behaviour (8–10). We used a variety of distance metrics and matching methods (propensity score, Mahalanobis distance without replacement, Mahalanobis distance with replacement) and found that both Mahalanobis methods were superior to the propensity score in producing comparison groups with matching variable distributions that were similar to those of the treatment (conservancy) group. We therefore used a Mahalanobis distance model with 1-to-1 nearest neighbour matching and replacement to create our comparison groups, implemented with the ‘Matching’ library of the statistical software R [14]. See references [11] and [15] for accessible treatments of matching issues.

**Figure 1:**
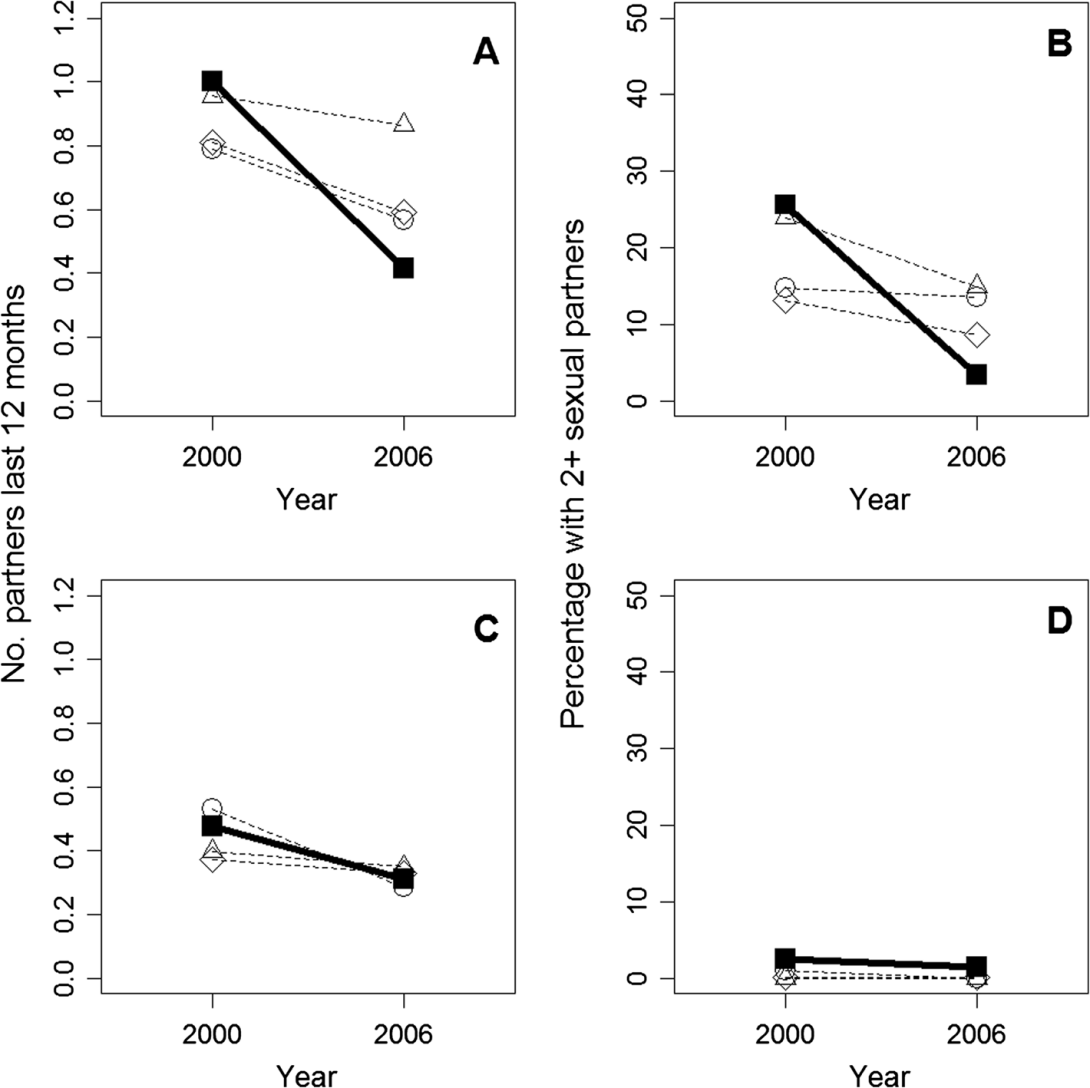
Changes from 2000 to 2006/2007 for conservancy residents (filled squares, solid line) versus 3 comparison groups (dashed lines, circles = quasi-experimental match; triangles = nearest geographical cluster; diamonds = entire non-conservancy population) for mean number of sexual partners over the last 12 months in (A) men and (C) women, and mean percentage having two or more sexual partners in the last 12 months in (B) men and (D) women.

We evaluated which comparison group had the smallest difference between conservancy and non-conservancy respondents in the number of sexual partners in 2000. For men the best comparison group was the nearest geographical cluster matching model, while for women it was the quasi-experimental matching model (for which good post-matching balance was achieved over all covariates). In both cases, this minimum difference was not statistically different from 0; i.e., numbers of sexual partners were statistically identical in conservancy and comparison groups in 2000. We then used these best matching models for men and women with the 2006/2007 data to produce a comparison group, composed of respondents with similar socio-demographic characteristics as comparison groups in 2000, for the statistical tests described below. This assumes that using the same matching model in both years would produce identical outcomes inside and outside conservancies in the absence of a program impact. Note however that results were qualitatively similar regardless of which particular matching model was used (Figure 1).

Our final dataset, covering both conservancy residents and matched non-residents, included 117 men and 318 women in 2000 and 170 men and 357 women in 2006/2007. We evaluated the statistical significance of the main effects of year and conservancy residence, and a conservancy-year interaction term, using generalized linear models. Response family was negative binomial for the (overdispersed) count of sexual partners, and binomial for the binary variable coding whether individuals had 2 or more sexual partners in the last year. The key test of whether the program had an impact on temporal trends in sexual partners was whether the conservancy-year interaction coefficient was significantly different than zero (Table 1).

**Table 1:** Regression model results for number of sexual partners and percentage having two or more partners, men and women

	***Men***				***Women***			
	**Coefficient**	**Std. Error**	***Z*-value**	***p***	**Coefficient**	**Std. Error**	***Z*-value**	***p***
***Number of sexual partners***								
Intercept	−0.044	0.151	−0.30	0.768	−0.744	0.10206	−7.289	< 0.001
Year	−0.100	0.200	−0.50	0.618	0.127	0.13997	0.907	0.364
Conservancy residence	0.044	0.187	0.24	0.812	−0.426	0.16214	−2.628	0.009
Year:conservancy interaction	−0.778	0.281	−2.76	0.006	−0.096	0.2255	−0.425	0.671
***% having 2+ sexual partners***								
Intercept	−1.158	0.346	−3.35	0.001	−3.674	0.4529	−8.112	< 0.001
Year	−0.583	0.487	−1.20	0.231	−0.526	0.7372	−0.713	0.476
Conservancy residence	0.091	0.428	0.21	0.832	0.912	0.5419	1.682	0.093
Year:conservancy interaction	−1.706	0.804	−2.12	0.034	−17.278	1244	−0.014	0.989

## Results

From 2000 to 2006/2007 there was a large (~ 60%) decrease in the mean reported number of sexual partners over the past 12 months for men in conservancies exposed to the community-based HIV/AIDS program. Mean number of partners in comparison groups also declined, but much less steeply, with men in the best comparison group showing a non-significant (~10%) decrease (Figure 1A, Table 1). Our data also show that the decrease in sexual partners is neither an incremental change in the distribution of those men having one partner versus none, nor a reflection of a few outlying individuals having large numbers of partners in 2000. Rather, it reflects a significant drop in the number of conservancy men having two or more sexual partners, relative to non-conservancy men (Figure 1B).

Numbers of sexual partners reported by women were lower than for men, with no program impacts on either mean number of partners (Figure 1C) or the mean number of women having two or more partners relative to comparison groups (Figure 1D).

## Discussion

HIV and AIDS outreach and policies associated with Namibia’s communal conservancy program have significantly reduced multiple sexual partnerships among men, arguably the main behavioural determinant of the disease’s spread in Africa [16-18]. With a reduction of approximately 50% relative to non-conservancy comparison groups, this result has important potential implications for reducing infections in communal areas of Namibia. We did not see the same impact among women, and suspect this is due to two factors: women in Namibia have lower numbers of partners than men and are much better-informed on issues of HIV and sexual health [7], and reported sexual partner data are less reliable for women, due to the stigma associated with accurately reporting multiple partners [10]. Given the high prevalence of HIV in sub-Saharan Africa and the devastating effects that the disease has on the social and economic fabric of communities, especially with regard to natural resource management, lessons from Namibia’s CBNRM program and the associated HIV/AIDS mainstreaming effort may help in slowing the disease in other communal areas of Africa. We also suggest quantitative evaluations of similar incipient programs [19,20] are urgently needed. These could improve on our study by designing prospective, experimental evaluations that collect new data tailored to purpose.

## Competing interests

The authors declare that they have no competing interests.

## Authors’ contributions

RN conceived the study, carried out the data analysis, and wrote the manuscript. KBJ carried out the data analysis and wrote the manuscript. Both authors have read and approved the final manuscript.
